# Indication of social buffering in disbudded calves

**DOI:** 10.1038/s41598-022-15919-8

**Published:** 2022-08-03

**Authors:** Katarína Bučková, Ágnes Moravcsíková, Radka Šárová, Radko Rajmon, Marek Špinka

**Affiliations:** 1 Department of Ethology, Institute of Animal Science, Přátelství 815, 104 00 Prague-Uhřínevěs, Czech Republic; 2 School of Biological Sciences, Institute for Global Food Security, Queen’s University Belfast, 19 Chlorine Gardens, BT9 5DL Belfast, Northern Ireland, UK; 3 Department of Ethology and Companion Animal Science, Faculty of Agrobiology, Food and Natural Resources, Czech University of Life Sciences, Kamýcká 129, 165 00 Prague 6, Czech Republic; 4 Department of Veterinary Sciences, Faculty of Agrobiology, Food and Natural Resources, Czech University of Life Sciences, Kamýcká 129, 165 00 Prague 6, Czech Republic; 5 Central Veterinary Administration of the State Veterinary Administration, Slezská 100/7, 120 00 Prague 2, Czech Republic

**Keywords:** Animal behaviour, Zoology

## Abstract

Most dairy calves are housed individually in early ontogeny but social housing has positive effects on calf welfare including an advantage of social buffering, i.e., when negative effects of stress are mitigated through social support of conspecific. The effects of social buffering has not yet been examined in relation to disbudding; a painful husbandry procedure commonly performed on young dairy calves. The objective of this study was to investigate the effect of pair versus individual housing on calves’ behavioral reaction to disbudding. In total 52 female calves were randomly allocated either to individual (n = 16) or pair housing (n = 36, 18 focal). Calves were hot-iron disbudded with a local anesthetic and their spontaneous behavior in home pens was recorded for 24 h pre- and post-disbudding. Eating forage, ruminating, resting, exploration, play, self-grooming, and pain-related behaviors were quantified during eight 20 min intervals during the 24 h periods pre- as well as post-disbudding. In pair-housed (PAIR) calves social resting, active and passive allo-grooming were additionally recorded. The differences between individually housed (INDI, n = 10) and PAIR calves (n = 12) were tested by general linear models. The changes in pre- and post-disbudding behaviors in all calves as well as in social behaviors of PAIR calves were tested by paired t-test. We found that head shaking (t =  − 3.46, P = 0.0024), head rubbing (t = 4.96, P < 0.0001) and self-grooming (t = 2.11, P = 0.04) increased in all calves after disbudding. Eating forage increased only in PAIR calves (t = 2.50, P = 0.030) which also resulted in a difference between treatments with PAIR calves fed more often than INDI calves (F_1,18_ = 12.96, P = 0.002). Differences in eating forage may be an indication of improved ability of PAIR calves to recover from disbudding. No other significant differences were detected between treatment groups which might have been caused by our limited sample. Our results provide the first evidence that housing treatment affects calves’ reactions to disbudding, with possible indication of social buffering.

## Introduction

Disbudding of calves is a common procedure in dairy farms^1^ which is defined as removal of horn buds in calves of up to 2 months of age whereas dehorning is defined as removal of horns in older cattle^2^. In the EU, 81% of dairy farms currently keep disbudded/dehorned animals^2^. In the Czech Republic, disbudding is performed in 93% of farms^3^. The main purpose of disbudding is the effort to decrease the risk of injuries among herdmates^2^. The most common method of bud removal is hot-iron disbudding^2,3^ where a bud growth is prevented through tissue cauterization^4^ producing severe pain^1^. Hot-iron disbudding wounds take 9 weeks to heal^5^ and calves experience ongoing pain for at least 3 weeks after the procedure^4^. Therefore, a combination of local anesthetic and a single dose of NSAID (non-steroidal anti-inflammatory drug) at the time of the procedure is currently the best ethical practice of mitigating pain after disbudding^4^. However, even if hot-iron disbudding is performed using anesthetic in a combination with NSAIDs, it is not possible to eliminate all negative effects on calf welfare^1,6,7^. For example, disbudded calves treated with anesthesia and NSAIDs show increased cortisol concentrations^1^ and decrease resting^6^ compared to non-disbudded calves.

In spite of this, any kind of medication for pain relief after and/or before disbudding is administered to the animals only in a small percentage of farms^2,3,8^. In the USA anesthetics/analgesics are used only by 28.2% of operations that disbud/dehorn heifer calves^8^. The situation in EU is similar as less than 30% of producers administer pain relief medication to the calves^2^. In the Czech Republic, more than 90% of producers do not report use of any pre- or post-procedure medication to mitigate the pain caused by disbudding^3^. Particularly the use of NSAIDs is established poorly, e.g., only 25% of Canadian cautery users report its use^9^. Therefore, animal welfare scientists should also consider other approaches to improve the welfare of disbudded calves, especially those which are not treated sufficiently before and/or after disbudding. One promising approach to improve calf welfare after disbudding is taking advantage of the effect of social buffering, the phenomenon by which the presence of affiliative social partners mitigates stress responses^10^.

Most dairy calves are separated from mothers shortly after birth^11^ and housed individually until 2 months of age^12,13^. However, there is an increasing interest in social housing of calves due to many positive effects on their development and welfare^14^. For example, social housing increases calf feed intake^15^, growth^16^ and play^16^. Recent studies showed that even housing calves with one peer leads to positive effects such as experiencing more positive affective states (i.e., the calf experiences its situation as more pleasant)^17^ or improved ability to cope with weaning^18,19^. Bolt et al. and De Paula Vieira reported that pair-housed calves cope with weaning stress better than individually housed calves as they vocalized less^18,19^, had shorter latencies to start feeding on starter^19^, visited the starter feeder more frequently^19^, spent more time in the feeder^19^ and consumed more solid feed^19^. Moreover, there are many findings across species that positive social interactions with a conspecific decrease negative effects of stress, e.g., facilitate wound healing^20–24^ or adverse consequences of maternal deprivation^25,26^.

However, there is no research addressing reaction to disbudding in individually versus socially housed calves. Therefore, the objective of this study was to investigate the effect of individual versus pair housing on calves’ behavioral reaction to disbudding. In relation to this, we hypothesized that compared to individually housed calves, pair-housed calves would cope with disbudding better due to the effect of social buffering. Specifically, we predicted that pair-housed calves would have a smaller decrease in those behaviors which indicate improved welfare after disbudding (i.e., in feeding, ruminating, resting, exploration, and play), but smaller increase in pain-related behaviors (i.e., head shaking, head rubbing, foot stamping, and self-grooming) than individually housed calves. It seems that feeding does not change after disbudding^27–30^, but Adcock and Tucker reported that disbudded calves spent more time suckling milk^31^, and cattle have been reported to decrease feeding after other stressful events, e.g., transportation^32,33^. The findings on ruminating and lying are also inconclusive as they were reported either to decrease^6,27,29^ or did not change after disbudding^30,34–36^. Exploration does not seem to change after disbudding^37^, but it may be decreased in other stressful situations, i.e., when exposed to a novel environment without a companion calf^38^. Play serves as an indicator of positive animal welfare which decreases in disbudded calves^35,39,40^. Head shaking, head rubbing, foot stamping and self-grooming are considered to be indicators of pain in disbudded calves^1,7,35,41–46^.

Moreover, it is not clear whether calves increase social contact to allow transmission of social buffering. Research on transmission of social buffering in cattle is rare, and the findings are not consistent. Ede et al. reported that calves spent more time in proximity and paid more attention to a conspecific in pain compared to a sham treated calf^47^. However, Gingerich et al. reported that disbudded calves left shelter more frequently when it was occupied^6^, and Turner et al. reported that ear tagging and castration did not result in calves receiving more maternal attention^48^. Therefore, our second objective was to investigate whether pair-housed calves increase social contact after disbudding. We hypothesized that due to their motivation to alleviate discomfort through social contact, pair-housed calves would show more social resting and more allo-grooming post- than pre-disbudding.

## Methods

### Animals and experimental procedures

The study was carried out at the Institute of Animal Science’s experimental farm Netluky in Prague, Czech Republic, from June 2017 to August 2018. For the randomized control trial, 52 Holstein Friesian female calves were used. Calves were separated from their mothers within 12 h after birth and housed individually until they entered the study at age 8.54 ± 1.96 days (mean ± SD). Sixteen calves were kept individually and 36 calves were housed in pairs. Animals were assigned to treatments randomly. Individually housed (INDI) calves were kept in standard single pens (1.4 × 2.6 m). They could have visual and tactile contact with calves in neighboring pens. Pair-housed (PAIR) calves were housed in double-sized pens (2.8 × 2.6 m). All pens were located in the same barn and bedded with straw. All calves had free access to water and calf concentrate as soon as they were separated from their mothers. They were checked for health daily by visual inspection and treated by a veterinarian if needed. In the first week of the experiment, calves were fed 7 l of milk per day via teat-buckets. In the second week, the amount of milk was increased to 8 l per day. From the third week onwards, calves were fed 10 l of milk per day and had free access to hay. Calves were fed twice a day during the first week of the experiment (at 6 a.m. and 6 p.m.) and three times a day during the remaining experimental period (additional feeding was scheduled for the midday). The calves were weighed 2 days pre-disbudding by two researchers familiar to them; their body weight (BW) was 86.1 kg ± 8.92 (mean ± SD). One individually housed calf was excluded during the course of the study due to impaired health and growth, and another individually housed calf was excluded shortly before disbudding as it showed signs of illness.

Disbudding was performed when calves were approximately 8 weeks old (exactly 58.84 ± 2.01 days, mean ± SD), i.e., in the last week of the study preferably on Thursday. Exceptionally, calves were disbudded another day (Friday: 6 calves, Saturday: 2 calves or Sunday: 8 calves). Because calves were entering the study gradually, there were multiple weeks of disbudding. Disbudding was performed by a trained veterinarian with the assistance of two researchers familiar to the calves. The calf was restricted in a dehorning crate in front of its home pen between 7 and 8 a.m. and 3 ml of 2% lidocaine were applied subcutaneously to cornual nerve of each horn bud using a 20 gauge needle. After 15 min an electric cautery iron (stainless steel), with a 1.8 cm tip and maximum declared temperature of 620 °C was applied to the horn bud for 15 s. Shortly after disbudding, aluminum spray was applied to each bud to protect it against external influences. Disbudding was performed in all experimental calves, i.e., also in non-focal pair-housed calves which were disbudded shortly before/after disbudding of their social partners.

### Data sampling and analysis

The calf behavior in a home-pen was recorded by camera system for 24 h pre- and post-disbudding. The camera system was composed of the network video recorder (DVR pro 4× AHD/TVI/CVI, Cantonk), HD cameras (2MPX AHD/TVI/CVI/CVBS, 1080P, IR LED 60 m, Cantonk), and the hard disk (SATA disk, 4T, CCTV). For observation, behaviors which may indicate improved welfare after disbudding (eating forage, ruminating, resting, exploration, play) and pain-related behaviors (head shaking, head rubbing, foot stamping, and self-grooming) were chosen. Those behaviors were analysed from the video recordings in 1 min intervals by one-zero sampling method (i.e., the behavior being present at any time during the 1-min interval)^49^ during first 20 min in 1, 2, 3, 4, 6, 9, 12 and 24 h periods pre- as well as post-disbudding. In PAIR calves social resting, active and passive allo-grooming were additionally observed. The definitions of all behaviors are given in Table 1. The sums of values were calculated for each behavior separately for pre-disbudding and post-disbudding period. The video recordings were assessed by a researcher experienced in work with dairy calves who achieved high intra-observer reliability in all behaviors (r > 98%; calculated by Pearson correlation coefficient from 8% of the final data set). If there was more than 25% of missing data, the calf was not included in the statistical analysis (INDI: n = 3, PAIR: n = 6). Data were not included in the analysis if there were missing values on BW which was used as an independent variable in the statistical models (INDI: n = 1). Thus, the final data set consisted of behavioral data from 10 INDI and 12 PAIR calves.

**Table 1:** The definition of behaviors observed in individually and pair-housed (PAIR) calves pre- and post-disbudding.

Behavior	Definition
Eating forage	Calf is taking hay or straw into the mouth followed by chewing and swallowing^50^
Ruminating	Calf is chewing after regurgitating^50^
Resting	Calf is lying in any resting position^50^
Exploration	Calf is sniffing walls or bedding^41^
Play	Individual—object play, gallop, jump, leap, buck-kick, head-shake, turn, Social—play fight, mount^51^ (observed only in PAIR calves)
Head shaking	Calf rapidly shakes its head from one side to the other^52^
Head rubbing	Calf lifts hind leg to scratch top of head with foot or rubs head against sides of the pen^43^
Foot stamping	Calf raises one foot and brings it down again immediately^43^
Self-grooming	Calf is licking itself^52^
Social resting	Calf is lying close to other calf^53^, i.e., they touch each other (observed only in PAIR calves)
Active allo-grooming	Calf is performing social licking (observed only in PAIR calves)
Passive allo-grooming	Calf is receiving social licking (observed only in PAIR calves)

The ethogram was based on the studies cited in the table.

The changes of the behaviors after disbudding were calculated as difference between post-and pre-disbudding behavior for the entire 24 h pre- and post-disbudding period. The differences between behaviors of INDI and PAIR calves were tested by general linear models (proc glm in SAS). Separate models were run for each dependent variable (i.e., each behavior). Housing treatment (INDI/PAIR), BW (kg) and the interaction between treatment and BW were included as fixed effects. BW values were centred for the analysis, i.e., the average was subtracted from value of each calf. Next, t-tests (proc ttest in SAS) were run to test if the behaviors significantly changed after disbudding, i.e., the post- minus pre-disbudding differences were compared to zero. If there was no significant effect of housing treatment, the t-test was run for all calves; if the effect of housing was significant, the behavior was analysed in two models, i.e., separately for INDI and PAIR calves. Plots of predicted values against residuals and distribution histograms of residuals were visually inspected to check the homoscedasticity and normality assumptions of all the general linear models. R^2^ was used as a goodness-of-fit measure. Data were analysed in SAS.

### Ethics declarations

This experiment was carried out in accordance with the ethical policy of the International Society of Applied Ethology. It was approved by the Institutional Animal Care and Use Committee of the Institute of Animal Science. The study is reported in accordance with ARRIVE guidelines.

## Results

### Housing effects on the reactions to disbudding

**Figure 1:**
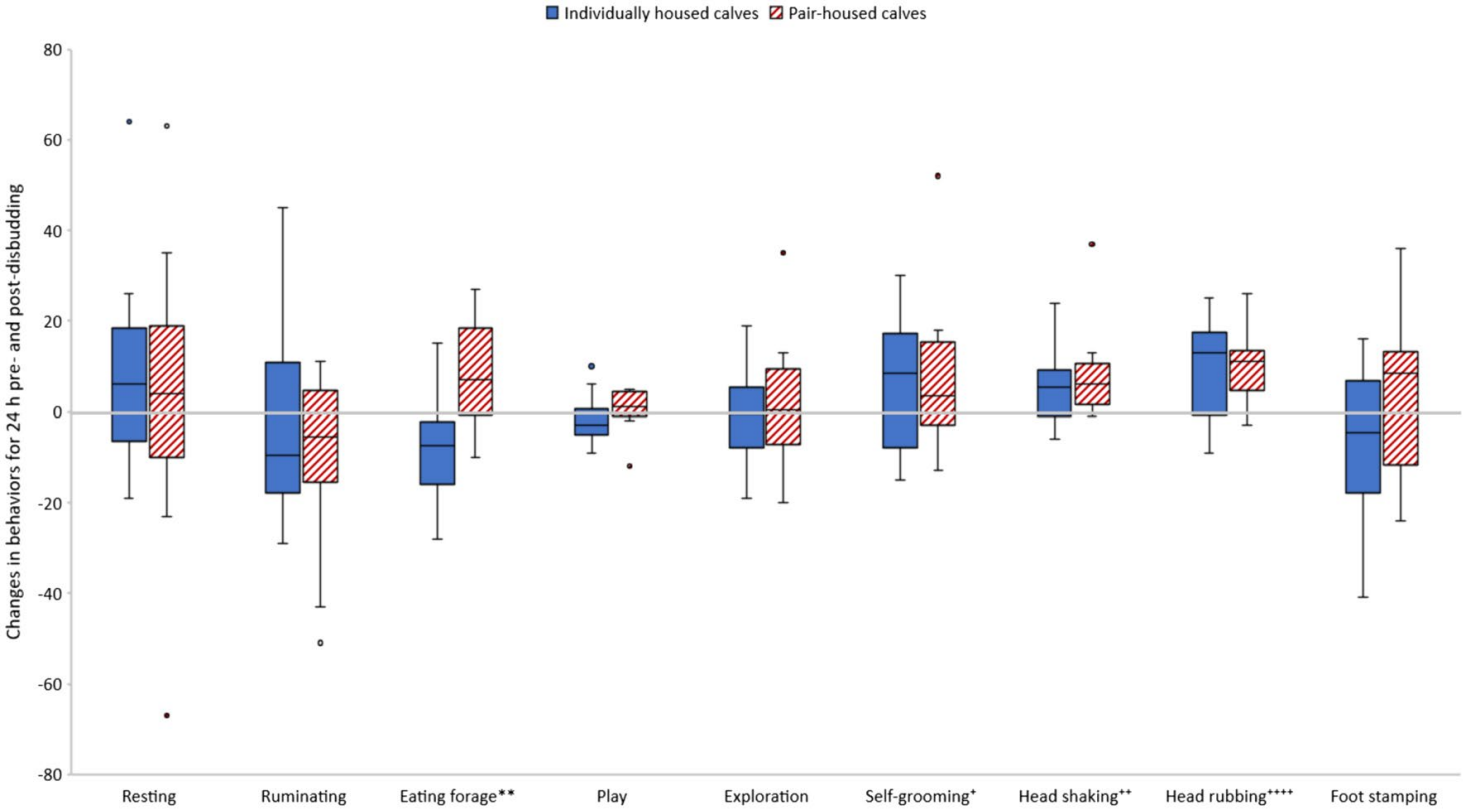
Changes in behaviors after disbudding calculated as difference between post-and pre-disbudding behavior. Disbudding significantly increased head-shaking, head-rubbing and self-grooming in all calves. Eating forage significantly increased only in PAIR calves which resulted in a significant difference between housing treatments. The boxplots depict median, interquartile range, data range as whiskers and outliers as circles. Blue boxes: Individually housed calves (n = 10). Hatched red boxes: Pair-housed calves (n = 12). Asterisks represent statistically significant differences between treatments (**P ≤ 0.01), crosses indicate statistically significant differences of all calves from zero, i.e., a significant change of the behavior after disbudding (^+^P ≤ 0.05, ^++^P ≤ 0.01, ^++++^P ≤ 0.0001).

Housing treatment had significant effect on feeding behavior change between post- and pre-disbudding as PAIR calves increased feeding post-disbudding whereas INDI calves decreased it (R^2^ = 0.51; PAIR: lsmeans = 8.23, confidence limits 1.65 to 14.81; INDI: lsmeans − 8.27, confidence limits − 15.49 to − 1.05; F_1,18_ = 12.60, P = 0.0023; the results are reported as lsmeans and confidence limits in all models mentioned in this paragraph). We did not detect significant differences between PAIR and INDI calves in resting behavior (R^2^ = 0.16; PAIR: 3.68, − 12.96 to 20.33; INDI: 9.93, − 8.33 to 28.19; F_1,18_ = 0.28, P = 0.60), ruminating (R^2^ = 0.11; PAIR: − 10.18, − 22.69 to 2.34; INDI − 2.50, − 16.23 to 11.23; F_1,18_ = 0.75, P = 0.40), exploration (R^2^ = 0.07; PAIR: 2.11, − 5.87 to 10.09; INDI − 0.17, − 8.86 to 8.66; F_1,18_ = 0.15, P = 0.70), play (R^2^ = 0.15; PAIR: 0.68, − 2.41 to 3.77; INDI − 1.64, − 5.03 to 1.75; F_1,18_ = 1.13, P = 0.30), and pain-related behaviors: head shaking (R^2^ = 0.04; PAIR: 8.12, 2.12 to 14.11; INDI 5.37, − 1.22 to 11.95; F_1,18_ = 0.42, P = 0.52), head rubbing (R^2^ = 0.12; PAIR: 10.58, 4.80 to 16.36; INDI 9.11, 2.77 to 15.46; F_1,18_ = 0.13, P = 0.72), foot stamping (R^2^ = 0.19; PAIR: 4.98, − 5.55 to 15.81; INDI − 6.54, − 18.09 to 5.01; F_1,18_ = 2.40, P = 0.14), and self-grooming (R^2^ = 0.09; PAIR: 7.64, − 2.08 to 17.35; INDI 6.61, − 4.05 to 17.27; F_1,18_ = 0.02, P = 0.88). Neither body weigh nor the interaction between body weight and treatment had influence on any of the dependent variables. The results on changes of post-disbudding behavior in individually and pair-housed calves are shown in Fig. 1.

### Changes in behavior after disbudding

Disbudding significantly increased self-grooming (mean post- minus pre-disbudding difference = 7.00, confidence limits 0.09 to 13.91, DF = 21, t = 2.11, P = 0.04; the results are reported as mean post- minus pre-disbudding difference and confidence limits in all models mentioned below), head shaking (6.86, 2.73 to 10.99, DF = 21, t = 3.46, P = 0.002) and head rubbing (9.95, 5.78 to 14.13, DF = 21, t = 4.96, P < 0.0001) in all calves. Eating forage significantly increased after disbudding only in PAIR calves (PAIR: 8.41, 0.99 to 15.83, DF = 11, t = 2.50, P = 0.030; INDI: − 8.308.22, − 16.84 to 0.24, DF = 9, t =  − 2.20, P = 0.06). Disbudding did not significantly change resting (6.05, − 6.27 to 18.36; DF = 21, t = 1.02, P = 0.32), ruminating (− 6.64, − 15.60 to 2.33; DF = 21, t =  − 1.54, P = 0.14), exploration (1.05, − 4.57 to 6.66; DF = 21, t = 0.39, P = 0.70), play − 0.32, − 2.59 to 1.95; DF = 21, t =  − 0.29, P = 0.77) and foot stamping (− 0.09, − 8.00 to 7.82; DF = 21, t =  − 0.02, P = 0.98) in our calves. The results on changes of post-disbudding behavior are shown in Fig. 1.

**Figure 2:**
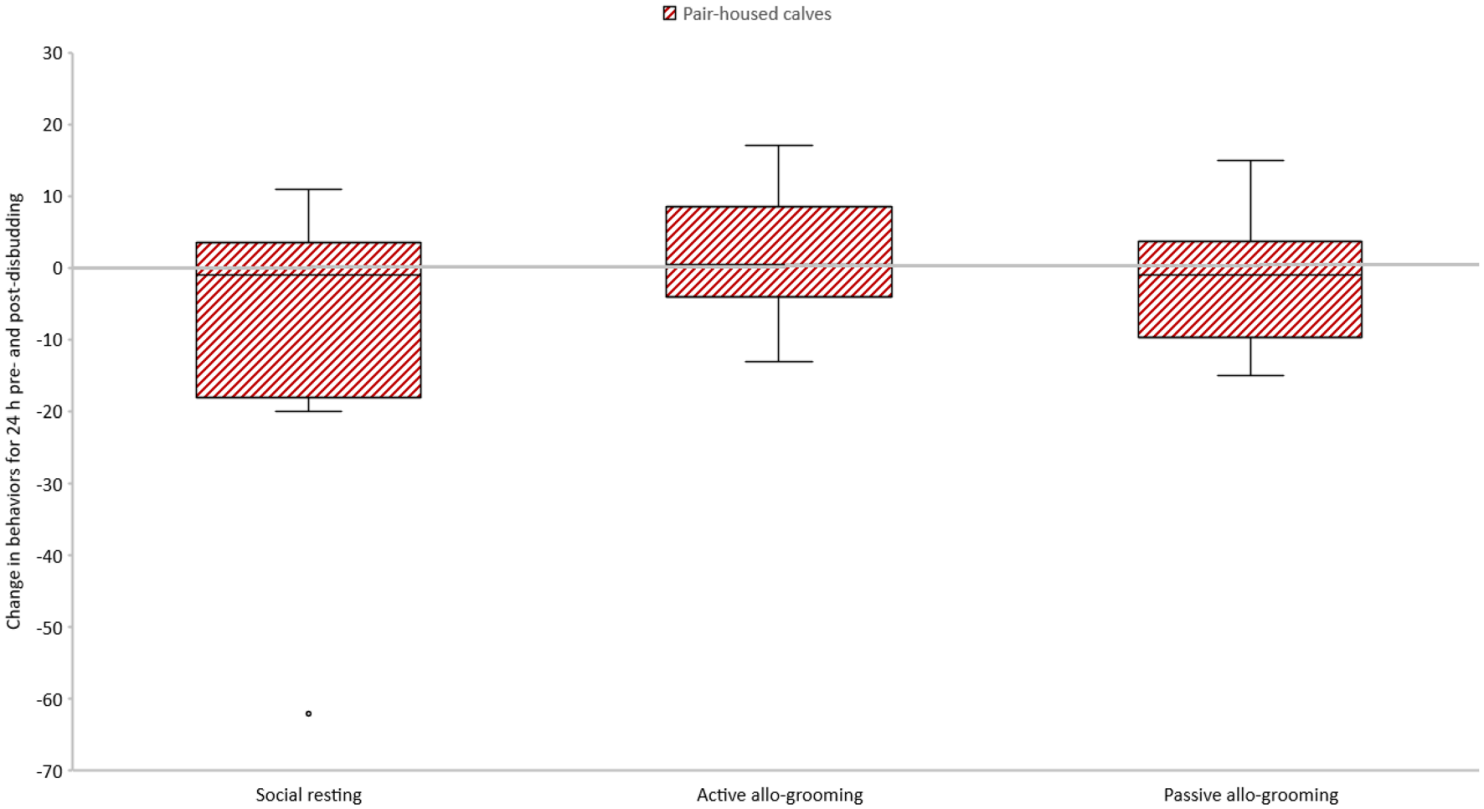
Changes in social behaviors in pair-housed calves (n = 12) calculated as difference between post-and pre-disbudding behavior. The boxplots depict median, interquartile range, data range as whiskers and outliers as circles.

Social behavior of PAIR calves did not differ between the pre- and post-disbudding periods (social resting: 11.58, − 27.37 to 4.20, DF 11, t =  − 1.62 P = 0.135; active licking: 1.50, − 3.81 to 6.81, DF 11, t = 0.62, P = 0.55; passive licking: − 1.75, − 7.31 to 3.81, DF 11, t =  − 0.69, P = 0.50). The results on behavior observed only in PAIR calves are shown in Fig. 2.

## Discussion

We aimed to compare the effect of individual versus pair housing on calves’ reaction to disbudding. Based on evidence of social buffering across species, we hypothesized that pair-housed calves would eat more forage, ruminate, rest, explore, and play more but decrease occurrence of pain-related behaviors after disbudding. We also hypothesized that pair-housed calves would show more social resting and allo-grooming post- than pre-disbudding.

We found that pair-housed calves increased eating forage (mostly hay as eating straw was observed less frequently) compared to individually housed calves after disbudding. It is well known that animals including cattle decrease feeding after experiencing stress^32,33,54–56^. Moreover, Theurer et al. reported that NSAID treated calves spent more time at the grain bunk compared to animals which were not treated with NSAID^57^, and Graf and Sen reported that calves disbudded with a local anesthetic spent more time feeding compared to calves disbudded without any pain relief medication^45^. Winder et al. also reported that sham-disbudded calves tended to drink milk sooner than disbudded calves^35^. Therefore, it is likely that individually housed calves experienced higher levels of stress caused by disbudding and thus decreased feeding. Conversely, the presence of a familiar social companion may have facilitated better recovery from disbudding. In this case, our finding would be the first evidence of social buffering in disbudded calves, consistent with the findings on social buffering in calves which were exposed to different types of stressors^18,19,38^. Bolt et al. and De Paula Vieira reported improved ability to cope with weaning stress in pair-housed calves compared to individually housed individuals^18,19^. Faerevik et al. reported that calves vocalized less, were more active and explored more when tested with a companion calf in a separation test^38^. Despite the more frequent eating forage, we did not find an increased ruminating in pair-housed calves. Non-significant changes in ruminating may indicate that more frequent eating does not necessarily mean that calves consume higher amount of forage.

Alternatively, Theurer et al. reported that calves treated with NSAIDs spent less time in the hay feeder^57^ and Adcock and Tucker reported that disbudded calves spent more time suckling milk due to its soothing effect or a need to meet increased energy requirements^31^. Thus, our disbudded pair-housed calves might have placed a higher value on eating forage due to a need to meet increased energy requirements and/or a soothing effect through oral manipulation, which would suggest the opposite, i.e., that pair-housed calves were affected by disbudding more. For example, calves might have been licking the wounds during social interactions which could have resulted in increased discomfort and/or pain. If so, our results would be more consistent with the findings of Gingerich et al. who reported that group-housed disbudded calves entered the shelter more frequently when it was not occupied and similarly left it more frequently when it was occupied. Authors explain their results by increased preference of disbudded calves for social withdrawal^6^ which have been reported in sick cattle^58,59^, and could be also another alternative explanation of our results.

There were no significant differences between treatments in resting, play, exploration, and pain-related behaviors. The explanation might be that these behaviors simply do not change between individually and pair-housed calves in relation to disbudding. Alternative explanation may be that social buffering sufficient for facilitation of recovering from disbudding was present in INDI calves, too. Our calves were housed in the same barn, so INDI calves could have visual, auditory and sometimes also (depending on occupation of adjacent pens) head-to head contact with other conspecifics.

We also did not detect any significant differences in social resting, active and passive allo-grooming observed in PAIR calves pre- and post-disbudding. Any of our negative results could have been due to the fact that we did not have sufficient sample size to detect these changes. This is reflected in the wide 95% confidence intervals that include both positive and negative values of the estimates. In some species (e.g., rats), tactile contact is an important cue for inducing social buffering^60^, and thus we could expect that positive social interactions occur more frequently after experiencing stress to allow transmission of social buffering. Our results are in contrast with findings of Ede et al. who reported that calves spent more time in proximity to a conspecific in pain^47^, and Gingerich et al. who even reported decreased interest of disbudded calves in sharing shelter with other calves^6^. Our results are consistent with the findings of Turner et al. who did not confirm increased maternal care towards calves displaying the most behavioral evidence of pain^48^. Taken together, further research is needed to investigate what quantity and which types of social interactions are effective for transmission of social buffering.

In future studies we encourage researchers to assess calf behavior for a longer period than 24 h as calves experience ongoing pain for at least 3 weeks following disbudding^4^. For example, it would be interesting to see how long the change in feeding persists. Furthermore, we suggest to use other methods (e.g., cognitive judgement bias task^61^) to assess welfare of disbudded calves in different housing conditions to further explore social buffering. Further research should also address effect of age and type of social bond on calf reaction to disbudding. Research on effect of social support in younger calves would be more applicable to farm practice because farmers prefer disbudding younger calves. For example, 63% of Czech farmers disbud calves before 4 weeks of age^3^. Our calves could not have been disbudded earlier as they were also used for the study where comparing affective states of non-disbudded individually and pair-housed calves was the main objective^17^. However, age may affect the existence and/or strength of social buffering among paired calves as the social bonds develop gradually in young cattle^62,63^. If calves are housed with the dam, a much stronger social bond is available for the calf than the bond to a peer in the case of pair-housing. The dam is the most preferred social partner during the first weeks of life, as she provides milk, active care and protection^64,65^. Therefore, it would be worthwhile to assess social buffering of maternal presence on disbudded calves.

Both a local anaesthetic and a NSAID should be administered to calves undergoing disbudding. However, our calves were treated with minimum amount of medication for pain relief as the objective of the study was particularly relevant to those calves which are not treated properly before disbudding. As the proper pain management in relation to disbudding is not yet established in the majority of dairy farms, other solutions are also needed to improve welfare of disbudded calves.

In conclusion, we did not detect a change in resting, ruminating, exploration, play and foot stamping in calves after disbudding. Neither did we detect differences between pair and individually housed calves in how they changed resting, ruminating, exploration, play, and pain-related behaviors from pre- to post-disbudding. We found no evidence for increased social contact among pair-housed calves in reaction to disbudding as the calves did not change their social resting or mutual allo-grooming behavior from pre- to post-disbudding. These negative results may have been partly due to the limited sample size in our study. However, three pain-related behaviors (head shaking, head rubbing and self-grooming) increased after disbudding in all calves which supports a growing body of evidence that both, anesthetic as well as non-steroidal anti-inflammatory drugs, should be administered to calves. Eating forage increased only in pair-housed calves after disbudding. Furthermore, it resulted in more frequent eating forage post-disbudding in pair-housed calves compared to individually housed animals. This finding is the first indication that socially housed calves may react to disbudding differently than individually housed calves which could be an indication of social buffering in disbudded dairy calves.

## Data Availability

The dataset generated during this study is available in the Figshare repository: https://figshare.com/articles/dataset/Data_on_indication_of_social_buffering_in_dairy_calves_xlsx/17159300.
